# The Association of Different Sedentary Patterns and Health-Related Physical Fitness in Pre-schoolers

**DOI:** 10.3389/fped.2021.796417

**Published:** 2022-01-03

**Authors:** Yanhua Lu, Yiyan Li, Tang Zhou, Menghao Sang, Longkai Li, Chunyi Fang, Wenwen Hu, Minghui Quan

**Affiliations:** ^1^School of Kinesiology, Shanghai University of Sport, Shanghai, China; ^2^Sports Department, Zhengzhou Business University, Zhengzhou, China; ^3^Dianfeng Fit Research Institute of Shanghai, Shanghai, China; ^4^Rehabilitation and Sports Medicine Research Institute of Zhejiang Province, Zhejiang Provincial People's Hospital, People's Hospital of Hangzhou Medical College, Hangzhou, China; ^5^Department of Physical Education, Institute of Disaster Prevention, Sanhe, China; ^6^Shanghai Frontiers Science Research Base of Exercise and Metabolic Health, Shanghai University of Sport, Shanghai, China

**Keywords:** health-related physical fitness, total sedentary time, ST Bouts time, ST Breaks times, pre-schoolers

## Abstract

**Background:** The results of sedentary time (ST) and health-related physical fitness (HPF) are not completely consistent and the studies concentrated on pre-schoolers are very limited.

**Methods:** We measured ST and ST patterns (ST Bouts time, ST Breaks times) by accelerometer. The health-related physical fitness T-score (HPFT) was calculated by five indexes: height-weight standard score, 20 m shuttle-run test, grip strength, standing long jump and 2 × 10 m shuttle-run test.

**Results:** We included 375 pre-schoolers (211 boys, 164 girls) in the final analysis. The total ST and ST Bouts times negatively correlated with HPFT in pre-schoolers. HPFT reduced by 1.69 and 0.70 points per 10 min increased in total ST and ST Bouts times, respectively. HPFT of the highest quartile group reduced by 9.85 points in total ST, and 10.54 points in ST Bouts time compared with the lowest quartile group. However, the HPFT increased by 0.09 points per 10 times increased in ST Breaks times; the HPFT increased by 16.21 and 15.59 points when moderate to vigorous physical activity (MVPA) replaced total ST and ST Bouts time.

**Conclusions:** HPF negatively correlated with the Total ST and ST Bouts times, but positively correlated with ST Breaks times; and HPF significantly improved when MVPA replaced ST in pre-schoolers.

## Introduction

Health-related physical fitness (HPF) is an essential indicator for evaluating the physical and mental health of human body, and good HPF is positively related to a person's cognitive development and academic performance (1, 2). Moreover, evidence shows that the HPF is also related to intellectual maturity in pre-schoolers (3). However, children's health problems keep coming up in recent years, such as the increased rate of obesity and weakened level of physique (4, 5). Thus, it is necessary to improve the level of HPF in young children.

Sedentary time (ST) is defined as behavior energy consumption < 1.5 METs when awake, such as sitting, leaning, or lying down postures (6). Although the results are not entirely consistent in studies that aimed to identify the relationship between ST and HPF in pre-schoolers (7, 8), most results showed that more ST was negatively related to HPF (9, 10). Therefore, the public health departments recommend that children under 5-year-old should have ST < 2 h and screen time within 1 h per day (11, 12). Besides, it was worth noting that ST patterns, such as ST prolonged for 20 min and the number of ST interruptions, may be related to HPF (13, 14).

The phenomenon of less movement not only exists in adults, but also in young children. Previous findings indicated that ST accounts for 34–94% of the daily awake time in pre-schoolers (15). Moreover, previous studies have shown that engaging in physical activity (PA) is beneficial for HPF (16), but few studies describe whether it can reduce the negative effects of ST accumulation on health when replacing ST with PA in pre-schoolers. At present, the relationship between ST, ST patterns and HPF is still inconsistent. Besides, most studies about the relationship between ST and HPF focused on children, adolescents, or the elderly. Research on pre-schoolers was still very limited.

Therefore, given shortcomings in the current study, this study aims to explore: (1) the relationship between total ST, ST patterns (Bouts time, Breaks times) and HPF in pre-schoolers and (2) whether the HPF level will change when ST is replaced by physical activity, based on the multiple linear regression and Isotemporal substitution model (ISM) in pre-schoolers. We hypothesize that lower HPF level correlates with longer total ST and ST Bouts times, and higher HPF level correlates with more ST Breaks times and physical activity time. The study results will provide evidence to determine the specific relationship between ST and HPF and give suggestions to improve HPF levels in pre-schoolers.

## Materials and Methods

### Participants

The study is cross-sectional research. The data were from the primary data of an observational study in 2013–2014 and a parallel intervention study in 2018. Both trials have been approved by the ethics review committee of Shanghai University of Sport, and has been registered in Chinese Clinical Trial Registration Center (Ethics approval number: 2015028, ChiCTR-OOC-15007439; Ethics approval number: 2017023, ChiCTR1900021552).

Inclusion criteria are: (1) pre-schoolers aged 3–6 years; (2) parents/guardians understood the whole experiment process and signed an informed consent form. Exclusion criteria are: (1) pre-schoolers who had a score < 75 points on the Wechsler Intelligence Scale; (2) who had cardiovascular or respiratory diseases; (3) who had been screened by a parent/guardian medical questionnaire and confirmed could not join in the moderate-intensity exercise.

**Figure 1:**
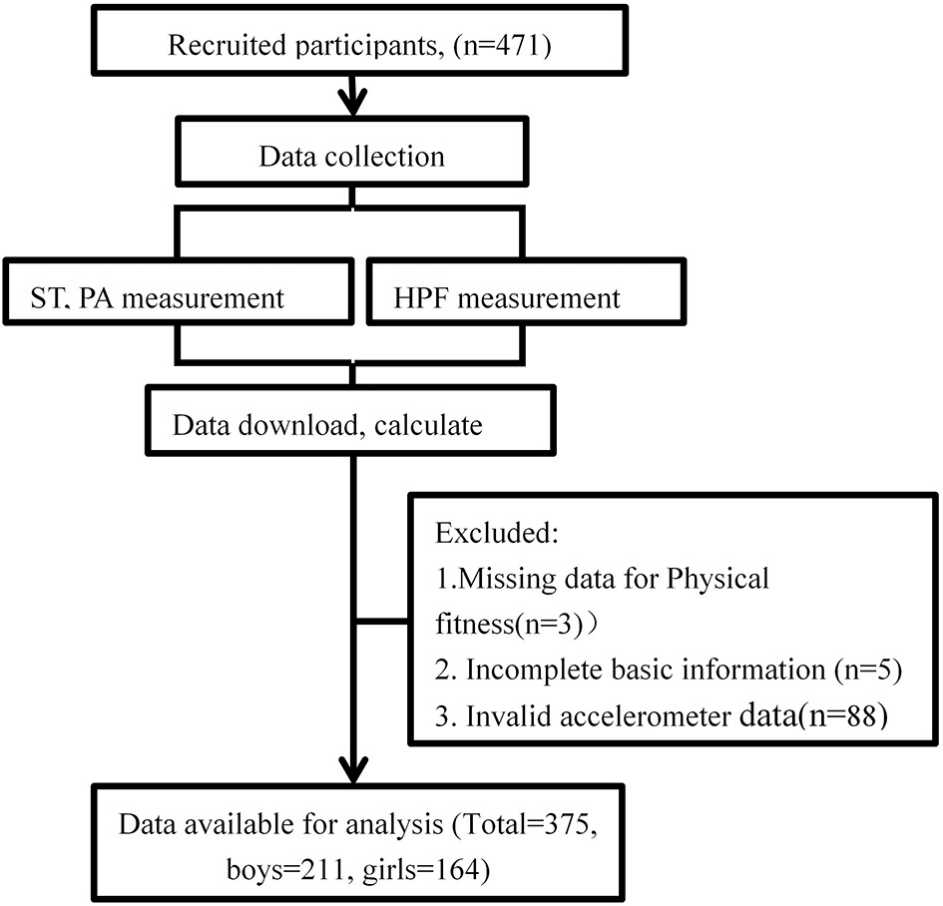
The flow of the participant in this study.

We recruited a total of 471 participants in the two trials, and 375 participants (211 boys, 164 girls; average age 4.56 years) were included in the final statistical analysis (Figure 1).

### Measurements

#### Basic Morphological Measurement and Family Status Survey

According to the Chinese National Physical Fitness Measurement Standards (Kindergarten Version), this study evaluates the height and weight of participants. The basic family status survey form was filled out by their parents, including the participant's sex, age, parents' education (Doctorate, Master, Undergraduate, Middle school and below), and monthly household income (below 2,000 yuan, 2,001–4,000 yuan, 4,001–8,000 yuan, 8,001–15,000 yuan, 15,001–30,000 yuan).

#### Total ST, ST Patterns and PA Measurement

ST and PA were measured by the three-axis accelerometer ActiGraph GT3X+ (Actigraph LLC, Pensacola, FL). The monitor has been proved effective to evaluate pre-schoolers' ST and PA (17). Participants need to wear GT3X+ for continuous 7 days to measure daily ST and PA (5 working days + 2 weekend days). The monitor was fixed on the waist belt and placed on the upper right hip, and it was required to be worn at all times except for bathing, swimming, and sleeping. The staff took back the monitor on the 8th day and used the Actilife software (Version 6.11.5) to download and view the data. We carried out the additional test for participants whose data did not meet the requirements or was missing after obtaining their parental consent.

The epoch was set to 1 s, and we processed ST, light physical activity (LPA), moderate to vigorous physical activity (MVPA) according to different cut points. The ST was defined using the cut-point of Evenson (<25 counts/15 s) (18), and Pate's cut-points were used to distinguish LPA (25 counts/15 s ≤ counts <420 counts/15 s) and MVPA (≥420 counts/15 s) (19). We defined the non-wearing time according to Choi's law (20), and the participants' valid data contained the complete data for at least 3 days (2 working days + 1 weekend day, effective daily wearing time was ≥480 min).

The definitions of ST Bouts times and ST Breaks times are related to the Epoch, and there is no standard for their purposes at present (13). We defined that each ST bouts must maintain at least 180 epochs (≥180 s), and 10% tolerance time was allowed during the process. An interruption lasts for at least one epoch or above (≥1 s) was considered an ST Breaks time.

#### Health-Related Physical Fitness Measurement

The HPF was assessed according to Chinese National Physical Fitness Measurement Standards (Kindergarten version). Given the importance of cardiorespiratory fitness to children's cognitive function, and quality of life and health showed in previous results; we added the evaluation of cardiorespiratory fitness to the HPF (21). Thus, the HPF of pre-schoolers included height-weight standard score, strength fitness (grip strength, standing long jump), cardiorespiratory fitness (20-meter shuttle-run test, 20 m-SRT) and speed-agility fitness (2 × 10 meters shuttle-run test, 2 × 10 m-SRT). The details of each testing item were described as below:

(1) According to the Chinese National Physical Fitness Measurement Standards (Kindergarten Version), we used the instruments of *Jianmin* brand (GMCS-I), which were designed instruments for Chinese National Physical Fitness Measurement when measuring the height and weight of participants, and to score in the same age and sex group.

(2) Strength fitness was evaluated by grip strength and standing long jump test. Before the grip strength test start, we adjusted the distance between the lower bar and the upper edge of the grip meter to be 4 cm, which had been proved to be a suitable distance for the pre-schoolers (22). Participants held a grip meter TKK-5401 (Takei, Niigata, Japan) with their dominant hand, standing vertically and naturally. The angle between the arm and body was about 10 degrees, and then held the bar as hard as possible until the value stopped changing. The test was conducted two times and we recorded the maximum value (accurate to 0.1 kg) as a result.

The staff made a demonstration first before the standing long jump test started. Participants stood naturally behind the start line, swung hands back and forth, bending knees, and then jumped forward in the air as far as possible. We used a tape to measure the straight-line distance from the start line to the participant's heel (accurate to 0.1 cm). We tested two times and recorded the best result.

(3) Cardiorespiratory fitness was evaluated by a 20 m-SRT. Participants ran to the rhythm of music from slow to fast between two lines separated 20 meters. The initial running speed was 8.5 km/h and increased by 0.5 km/h per 1 min. The test stopped when the participant could not step on or step over the end line within the specified time for two consecutive times, or felt unable to accomplish after repeated encouragement. An adult tester led the entire test because the participants were too young to understand the rules and requirements of the test thoroughly. We measured once and recorded the number of finished laps (20 meters is one lap).

(4) This study applied 2 × 10 m-SRT to evaluate the speed-agility fitness of pre-schoolers. Participants ran straight along two lines, 10 meters apart. Participants touched the marker when reached the finishing line, and then returned to the starting line. We recorded, using a stopwatch, the time from when the participants started to when they returned to the starting line. Participants accompanied an adult tester to ensure the test was successful. We tested two times and recorded the fastest time as result (accurate to 0.01 s).

### Data Analysis

We used the mean (M), standard deviation (SD), the frequency and percentage (*N*, %) to describe the distribution of continuous and categorical variables. The formula of standard *T*-score was: *T*=50+10^*^(X-M)/SD, X is a personal performance. Besides, we multiplied the results of the 2 × 10 m-SRT by −1 when calculating the T-score, because the smaller the value was, the better the result would be. The HPFT was the sum of the standard T-score of five indexes in different sex groups. HPFT = T_Height−weight standard score_ + T_20 m−SRT_ + T_2 × 10*m*−*SRT*_ + T_Standing long jump_ + T_Grip_.

A multiple linear regression model was used to explore the relationship between total ST, ST patterns and HPFT in pre-schoolers. Total ST, ST Bouts times were standardized for the total behavior time using the residual method (23). Similarly, ST Breaks times was standardized for the total ST in the same way. We put the total ST and ST patterns into the linear regression model as continuous variables and quartile groups (Q1–Q4) two forms, and HPFT of pre-schoolers is regarded as dependent variables. We put the median of each quartile groups (Q1–Q4) as a new variable into the regression model to obtain the trend test results. The model was adjusted for sex, age, household income, father's and mother's education.

The ISM was used to explore the change of outcome when a behavior replaced another behavior, and it has been used as an effective, simple model in epidemiological research (24). We used ISM to explore the HPF change when 30-min ST, and ST Bouts times were replaced by MVPA in pre-schoolers, while keeping other activities time consistent.

Additionally, the piece-wise linear regression model was used to explore whether there was a non-linear relationship between the ST patterns and HPFT after adjusting for confounding factors.

SPSS 21.0 and Empower software based on R language were used to analyze data; we consider it is statistically significant if *P* < 0.05.

## Results

We found girls were higher than boys in ST Bouts time, but inverse in ST Breaks times. It indicated that boys are more active than girls in pre-schoolers (Table 1). Besides, the results of multiple linear regression and the ISM analysis have no interaction between gender, so all the data are combined for analysis (Tables 2, 3).

**Table 1:** Basic information of the participants.

	**Boys** **(***n*** = 211)**	**Girls** **(***n*** = 164)**	**Total** **(***n*** = 375)**
**Anthropometric**			
Age (y)	4.58 ± 0.46	4.53 ± 0.47	4.56 ± 0.46
Height (cm)	111.63 ± 4.88	110.07 ± 4.92	110.95 ± 4.95^*^
Weight (kg)	20.64 ± 3.52	19.06 ± 2.73	19.95 ± 3.29^*^
**Sedentary behavior**			
Total ST (min/d)	576.49 ± 28.74	589.13 ± 27.20	580.19 ± 64.01
ST Bouts time (min/d)	394.61 ± 69.80	412.75 ± 71.00	402.52 ± 70.81^*^
ST Breaks times (times/d)	2,439.64 ± 401.31	2,287.55 ± 347.71	2,373.36 ± 390.10^*^
**Physical activity**			
LPA (min/d)	99.10 ± 18.06	91.79 ± 16.58	95.90 ± 17.78^*^
MVPA (min/d)	73.50 ± 18.37	66.67 ± 15.05	70.52 ± 17.31^*^
Total behavior time	749.11 ± 74.85	743.88 ± 66.93	746.82 ± 71.44
**Health-related physical fitness**			
Standing long jump (cm)	85.62 ± 16.51	82.27 ± 16.01	84.16 ± 16.35^*^
Grip strength (kg)	6.95 ± 2.33	6.04 ± 2.26	6.55 ± 2.34^*^
2 × 10 m shuttle-run test (s)	7.25 ± 0.93	7.34 ± 0.77	7.29 ± 0.86
20 m shuttle-run test (laps)	12.43 ± 4.56	12.91 ± 4.92	12.64 ± 4.72
HPFT	250.91 ± 32.56	248.83 ± 30.71	249.97 ± 31.96
**Socioeconomic status**			
**Father's degree**			
Less than middle school	7 (3.35%)	8 (4.91%)	15 (4.03%)
Middle school	26 (12.44%)	22 (13.50%)	48 (12.90%)
High school	51 (24.40%)	41 (25.15%)	92 (24.73%)
Undergraduate	73 (34.93%)	55 (33.74%)	128 (34.41%)
Master	41 (19.62%)	26 (15.95%)	67 (18.01%)
Doctor	11 (5.26%)	11 (6.75%)	22 (5.91%)
**Mother's degree**			
Less than middle school	4 (1.92%)	7 (4.29%)	11 (2.96%)
Middle school	35 (16.83%)	19 (11.66%)	54 (14.56%)
High school	55 (26.44%)	52 (31.90%)	107 (28.84%)
Undergraduate	73 (35.10%)	49 (30.06%)	122 (32.88%)
Master	30 (14.42%)	27 (16.56%)	57 (15.36%)
Doctor	11 (5.29%)	9 (5.52%)	20 (5.39%)
**Household-income (RMB/m)**			
Under 2,000	2 (0.97%)	7 (4.32%)	9 (2.44%)
2,000–4,000	11 (5.31%)	6 (3.70%)	17 (4.61%)
4,001–8,000	44 (21.26%)	33 (20.37%)	77 (20.87%)
8,001–15,000	87 (42.03%)	69 (42.59%)	156 (42.28%)
15,001–30,000	55 (26.57%)	36 (22.22%)	91 (24.66%)

*ST, sedentary time; LPA, light physical activity; MVPA, moderate to vigorous physical activity; Total behavior time, ST + LPA + MVPA; HPFT, Health-related physical fitness T-score. Data was described by Mean ± SD or N (%)*;

“^*^” means P for sex < 0.05.

**Table 2:** The association between ST patterns and HPFT.

		**HPFT (β, 95% CI)**	
	**Model 1** (***n*** **=375)**	**Model 2** (***n*** **=371)**	**Model 3** (***n*** **=369)**
**Total ST (per 10 min/d)**	**−1.62 (−2.73, −0.50)**	**−1.72 (−2.77, −0.67)**	**−1.69 (−2.78, −0.60)**
**Total ST quartile (min/d)**			
Q1 (394.73–534.65)	0 (ref)	0 (ref)	0 (ref)
Q2 (534.66–582.06)	0.23 (−8.86, 9.33)	−1.40 (−9.78, 6.99)	0.55 (−7.99, 9.09)
Q3 (582.07–623.79)	**−9.55 (−18.62, −0.48)**	**−10.85 (−19.26, −2.44)**	**−9.09 (−17.70, −0.49)**
Q4 (623.80–838.97)	**−9.36 (−18.43, −0.29)**	**−9.74 (−18.24, −1.24)**	**−9.85 (−18.58, −1.11)**
*P* for trend	**0.01**	**<0.01**	**<0.01**
**ST Bouts time (per 10 min/d)**	**−0.69 (−1.21, −0.17)**	**−0.69 (−1.18, −0.20)**	**−0.70 (−1.20, −0.20)**
**ST Bouts Time quartile (min/d)**			
Q1 (202.01–354.51)	0 (ref)	0 (ref)	0 (ref)
Q2 (354.52–398.41)	1.51 (−7.55, 10.57)	1.22 (−7.15, 9.60)	3.79 (−4.76, 12.34)
Q3 (398.42–446.27)	**−9.87 (−18.93, −0.81)**	−8.12 (−16.54, 0.30)	**−7.27 (−15.80, 1.25)**
Q4 (446.28–650.81)	**−11.44 (−20.48, −2.41)**	**−11.16 (−19.56, −2.75)**	**−10.54 (−19.20, −1.89)**
*P* for trend	**<0.01**	**<0.01**	**<0.01**
**ST Breaks times (per 10 times/d)**	**0.10 (0.01, 0.18)**	**0.09 (0.01, 0.17)**	**0.09 (0.004, 0.17)**
**ST Breaks times quartile (times/d)**			
Q1 (1,339–2,094)	0 (ref)	0 (ref)	0 (ref)
Q2 (2,095–2,392)	6.73 (−2.43, 15.88)	9.16 (0.77, 17.56)	7.75 (−0.85, 16.36)
Q3 (2,393–2,640)	7.89 (−1.26, 17.05)	7.39 (−1.01, 15.80)	6.49 (−2.17, 15.16)
Q4 (2,641–3,411)	**9.32 (0.19, 18.45)**	**9.30 (0.80, 17.80)**	7.92 (−0.84, 16.69)
*P* for trend	0.05	0.06	0.11

*Model 1: no adjusting*.

*Model 2: adjusting for age, sex*.

*Model 3: adjusting for age, sex, father's education, mother's education, household income*.

*The bold values were statistically significant*.

**Table 3:** The ISM results of MVPA and HPFT (30 min/d).

	**HPFT** (***β*****, 95% CI)**	
**MVPA**	**Model 1**	**Model 2**
Replace total ST	19.59 (12.35, 26.84)	16.21 (9.35, 23.07)
Replace ST Bouts time	18.47 (11.03, 25.92)	15.59 (8.57, 22.61)

*Model 1: adjusting for total behavior time*.

*Model 2: adjusting for total behavior time, age, sex, father's education, mother's education, household income*.

Results showed total ST and ST Bouts time negatively correlated with HPFT after adjusting for confounding factors such as sex, age, household income, father's and mother's education. HPFT reduced by 1.69 points (95% CI = −2.78, −0.60) and 0.70 points (95% CI = −1.20, −0.20) per 10 min increased in total ST and ST Bouts time, respectively. HPFT of highest quartile group of children reduced by 9.85 points (95% CI = −18.58, −1.11) in total ST, and 10.54 points (95% CI = −19.20, −1.89) in ST Bouts time compared with the lowest quartile group, respectively. However, the HPFT increased by 0.09 points (95% CI = 0.004, 0.17) when ST Breaks times increased per 10 times (Table 2).

Moreover, HPFT significantly increased by 16.21 points (95% CI = 9.35, 23.07) and 15.59 points (95% CI = 8.57, 22.61), respectively, when 30-min MVPA replaced the total ST, ST Bouts time and kept other activities time unchanged (Table 3).

Finally, after adjusting for confounding factors, no non-linear relationship was observed between ST patterns and HPFT.

## Discussion

### Main Finding

The study aims to explore the relationship between total ST, ST patterns and HPFT in pre-schoolers. We found the total ST and ST Bouts times negatively correlated with HPFT, and ST Breaks times positively correlated with HPFT after adjusted for confounding. Besides, HPFT increased significantly when 30-min MVPA replaced total ST and ST Bouts time in ISM.

### Comparison With Similar Research

This study showed a negative correlation between total ST, ST Bouts time and HPFT, which was consistent with most previous studies. Findings showed that individuals with more ST had a higher risk of central obesity, a higher weight value, and ST was also negatively related to cardiorespiratory fitness (9, 25, 26); and ST Bouts time negatively correlated with health indicator among adolescents (27). However, some studies have not found that the total ST and its change trajectory were related to HPF and body composition in pre-schoolers (28, 29); and results also showed no relationship between total ST and risk of cardiovascular disease, or cardiorespiratory fitness in children and adolescents (30, 31). The reasons for inconsistent results may be as follows: (1) Different tools were used to measure ST. Questionnaires, parental reports (8) or objective accelerometer (7, 29) were used in studies. Findings showed a big difference between subjective and objective ST, and the time measured by objective tools was closer to the actual value (32). (2) The parameter settings are different when measuring ST. The rules for including data, such as valid days were used at least 3 days (8) or 1 day (33) in different studies. (3) Participants accumulated different total ST in studies. The total ST of pre-schoolers may not reach the threshold that could affect the relationship between itself and HPFT in some studies (7, 28). Thus, given the possible shortcomings of previous studies, we choose the accelerometer to measure the total ST and selected valid data for at least 3 days in the final statistical analysis, to ensure the measured value was closer to participants' actual state. Furthermore, this study further analyzed and found a significant negative correlation between ST Bouts time and HPFT in Pre-schoolers, thus further revealing the relationship between different ST patterns and HPFT.

In addition, our results were consistent with previous studies that the more ST Breaks times, the higher HPFT in pre-schoolers. Some scholars had proposed increasing ST Breaks times as a method to change people's long-term sitting position, and to prevent health problems caused by ST a long time ago (34). Studies have found that ST Breaks positively impacted the health in children and adolescents (35). Even a simple standing interrupting behavior could induce beneficial metabolic changes, such as making insulin, total cholesterol content and fast blood sugar in an inevitable decline (36). Besides, findings were also observed that ST Breaks times could significantly improve the body's glucose metabolism with no increase in total energy intake among overweight and obese children (37), and positively reduce postprandial hypoglycemia and insulin responses among adults (38). The result seems to provide an effective way to improve the overweight state of children and adolescents. According to the positive correlation between ST Breaks times and HPFT, we conjecture that ST Breaks times may be related to PA. Pre-schoolers may improve HPF by performing PA during the ST Breaks. So far, most of the previous studies which identified the relationship between ST patterns and HPFT focused on children or adolescents (13, 14, 23). Obviously, findings of this study provide additional evidence to this research field.

Otherwise, we used the ISM to explore whether PA can attenuate the antagonistic relation between ST and HPFT. Results showed that the HPFT of pre-schoolers significantly increased when 30-min MVPA replaced total ST and ST Bouts time. It was also in line with our initial hypothesis and consistent with studies on adolescents (39). Does replacing the ST with any intensity of PA have such an effect? We found no similar change when replacing 30-min ST with LPA as with MVPA. In previous studies, the ISM was rarely used to explore the relationship among PA, ST and physical fitness in pre-schoolers. Our findings showed that the relationship between MVPA and HPFT was closer than that between LPA and HPFT. It indicated that accumulating more MVPA might be an appropriate way to improve physical fitness, which was consistent with the recommendation of at least 60 min per day of MVPA in pre-schoolers (11, 12). Unfortunately, only a small percentage of pre-schoolers met the age-specific recommendations of PA. For instance, only 13.7% of pre-schoolers reached at least 60 min per day of MVPA recommendation in a Canadian study (40). Moreover, a meta-analysis covered 6,309 pre-schoolers (from 29 articles in 7 countries) reported that the average daily MVPA was only 42.8 min (95% CI: 28.9–56.8) (41). In short, this study comprehensively considers the relationship between total ST, ST patterns and HPF in pre-schoolers. Results provide support for improving the HPF of pre-schoolers by controlling ST.

### Strengths and Limitations

There are some advantages in this study: (1) The study includes total ST and ST patterns, which show the relationship between the variables more realistically; (2) The study is carried out in the pre-schoolers, enriched research evidence of different populations; (3) The study combines multiple linear regression with the ISM to explore the relationship, made the results more reliable. There are also some limitations: (1) The participants are regional and the results cannot represent that in other ethnic, regional populations; (2) The study is cross-sectional research, which can only reflect the relationship between ST and pre-schoolers at the time point, cannot explain the cause and effect relationship; (3) The accelerometer fails to distinguish the posture of the participants' ST, and this is a restriction in monitoring the upper limb activities, but the accelerometer is the most accurate and standard tool to measure ST at present.

### Recommendations for Future Research

We suggest more detailed ST patterns research can be carried out in future, for example, (1) Dividing ST Bouts time into different phases (1–10 min, >10 min); (2) Carrying out more longitudinal and experimental studies to explore the cause and effect relationship between ST patterns and HPFT; (3) Considering the interaction of ST, PA and sleep on health indicators and optimal time allocation plan.

## Conclusions

Total ST, ST Bouts times negatively correlated with HPF, and ST Breaks times positively correlated with HPF in pre-schoolers. Besides, HPF significantly improved when MVPA replaced ST. We should decrease the ST and increase ST Breaks times to promote good physical development in pre-schoolers.

## Data Availability Statement

The raw data supporting the conclusions of this article will be made available by the authors, without undue reservation.

## Ethics Statement

The studies involving human participants were reviewed and approved by the Ethics Review Committee of Shanghai University of Sport (2015028, 2017023). Written informed consent to participate in this study was provided by the participants' legal guardian/next of kin.

## Author Contributions

MQ, YLu, TZ, MS, LL, CF, and WH: substantial contributions to conception and design, acquisition of data, or analysis and interpretation of data. YLu, YLi, and MQ: drafting the article or revising it critically for important intellectual content. All authors: final approval of the version to be published.

## Funding

This research was funded by the National Key Research and Development Program of China (2020YFC2003301, 2020YFC2007005), the National Natural Science Foundation of China (81703252), and Shanghai Key Lab of Human Performance (Shanghai University of Sport) (No. 11DZ2261100).

## Conflict of Interest

The authors declare that the research was conducted in the absence of any commercial or financial relationships that could be construed as a potential conflict of interest.

## Publisher's Note

All claims expressed in this article are solely those of the authors and do not necessarily represent those of their affiliated organizations, or those of the publisher, the editors and the reviewers. Any product that may be evaluated in this article, or claim that may be made by its manufacturer, is not guaranteed or endorsed by the publisher.
